# Long-term efficacy and safety of alemtuzumab in patients with RRMS: 12-year follow-up of CAMMS223

**DOI:** 10.1007/s00415-020-09983-1

**Published:** 2020-06-24

**Authors:** Brian Steingo, Yaser Al Malik, Ann D. Bass, Regina Berkovich, Matthew Carraro, Óscar Fernández, Carolina Ionete, Luca Massacesi, Sven G. Meuth, Dimos D. Mitsikostas, Gabriel Pardo, Renata Faria Simm, Anthony Traboulsee, Zia Choudhry, Nadia Daizadeh, D. Alastair S. Compston, the CAMMS223, CAMMS03409, and TOPAZ Investigators

**Affiliations:** 1 Infinity Clinical Research, Sunrise, FL USA; 2 King Saud Bin Abdulaziz University for Health Sciences, King Abdulaziz Medical City, Riyadh, Saudi Arabia; 3 Neurology Center of San Antonio, San Antonio, TX USA; 4 Keck School of Medicine of University of Southern California, Los Angeles, CA USA; 5 Synergy Healthcare Medical Associates, Los Angeles, CA USA; 6 Novant Health, Charlotte, NC USA; 7 Fundación IMABIS, Hospital Universitario Carlos Haya, Málaga, Spain; 8 University of Massachusetts Memorial Medical Center, Worcester, MA USA; 9 Department of Neurosciences, Drugs and Child Health, University of Florence, Florence, Italy; 10 Clinic of Neurology with Institute of Translational Neurology, University Clinic Münster, Münster, Germany; 11 First Neurology Department, Aeginition Hospital, National and Kapodistrian University of Athens, Athens, Greece; 12 Oklahoma Medical Research Foundation, Oklahoma City, OK USA; 13 Universidade de São Paulo, São Paulo, Brazil; 14 University of British Columbia, Vancouver, BC Canada; 15 Sanofi, Cambridge, MA USA; 16 University of Cambridge, Cambridge, UK

**Keywords:** Alemtuzumab, Multiple sclerosis, Disease-modifying therapy, Efficacy, Safety, Long-term

## Abstract

**Background:**

In the phase 2 CAMMS223 trial (NCT00050778), alemtuzumab significantly improved clinical and MRI outcomes versus subcutaneous interferon beta-1a over 3 years in treatment-naive patients with relapsing–remitting MS. Here, we assess efficacy and safety of alemtuzumab over 12 years in CAMMS223 patients who enrolled in the CAMMS03409 extension (NCT00930553), with available follow-up through the subsequent TOPAZ extension (NCT02255656).

**Methods:**

In CAMMS223, patients received 2 alemtuzumab courses (12 mg/day; baseline: 5 days; 12 months later: 3 days); 22% received a third course. In the open-label, nonrandomized extensions, patients could receive as-needed additional alemtuzumab or other disease-modifying therapies.

**Results:**

Of 108 alemtuzumab-treated patients in CAMMS223, 60 entered the CAMMS03409 extension; 33% received a total of 2 alemtuzumab courses, and 73% received no more than 3 courses through Year 12. Over 12 years, annualized relapse rate was 0.09, 71% of patients had stable or improved Expanded Disability Status Scale scores, and 69% were free of 6-month confirmed disability worsening. In Year 12, 73% of patients were free of MRI disease activity. Cumulatively throughout the extensions (Years 7–12), 34% of patients had no evidence of disease activity. Adverse event (AE) incidence declined through Year 12. Infusion-associated reactions peaked at first course and declined thereafter. Cumulative thyroid AE incidence was 50%; one immune thrombocytopenia event occurred, and there were no autoimmune nephropathy cases.

**Conclusions:**

Alemtuzumab efficacy was maintained over 12 years in CAMMS223 patients, with 73% receiving no more than three courses. The safety profile in this cohort was consistent with other alemtuzumab clinical trials.

**Electronic supplementary material:**

The online version of this article (10.1007/s00415-020-09983-1) contains supplementary material, which is available to authorized users.

## Introduction

Multiple sclerosis (MS) is a lifelong potentially debilitating disorder of the central nervous system that usually evolves over several decades and requires long-term treatment to slow the accumulation of disability and disease progression [1–3]. Alemtuzumab (LEMTRADA^®^; Sanofi, Cambridge, MA, USA) is an anti-CD52 humanized monoclonal antibody approved for the treatment of relapsing–remitting MS (RRMS) [4, 5]. In a 3-year phase 2 trial (CAMMS223; NCT00050778), patients with active RRMS who were treatment-naive at baseline demonstrated significantly greater improvements in clinical and radiological outcomes with alemtuzumab compared with subcutaneous interferon beta-1a (SC IFNB-1a; Rebif^®^; EMD Serono Inc., Rockland, MA, USA) [6]. Alemtuzumab also demonstrated significant efficacy versus SC IFNB-1a over 2 years in phase 3 clinical trials (CARE-MS I [NCT00530348] and II [NCT00548405]) [7, 8], and efficacy was maintained in two consecutive extension studies (CAMMS03409 [NCT00930553]; TOPAZ [NCT02255656]) [9–13]. Adverse events (AEs) associated with alemtuzumab in clinical trials and post-marketing experience include infusion-associated reactions (IARs), increased frequency of infection and the potential for opportunistic infections, secondary autoimmunity (thyroid disorders, immune thrombocytopenia [ITP], nephropathies, autoimmune cytopenias, autoimmune hepatitis, and other less common autoimmune events), acute acalculous cholecystitis, and cardiovascular and pulmonary events possibly related to infusion [4, 6–11, 14–16].

Alemtuzumab-treated patients from the CAMMS223 study could participate in a follow-up period of 2.5 additional years on average, and then enroll in the CAMMS03409 and subsequent TOPAZ extension studies. This long-term follow-up period allowed an assessment of efficacy and safety outcomes in alemtuzumab-treated CAMMS223 patients over 12 years.

## Methods

### Study design, participants, and procedures

The study designs of the phase 2 CAMMS223 trial, and the CAMMS03409 and TOPAZ extension studies have previously been described [6, 9–13, 17].

#### CAMMS223 study

**Fig. 1:**
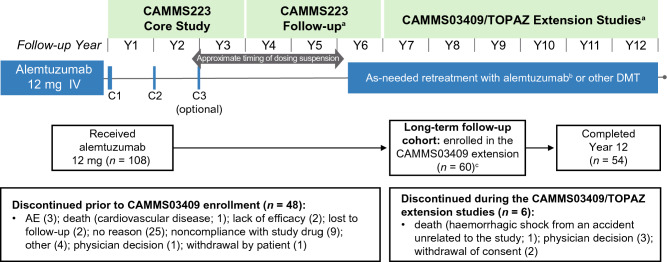
Study design and patient disposition of the CAMMS223 long-term follow-up cohort. Schematic of the CAMMS223 long-term follow-up cohort participation (*N* = 60) from the core CAMMS223 trial through the CAMMS03409 extension study and TOPAZ. For each patient of the long-term follow-up cohort, data were analyzed from enrollment in the CAMMS223 core study until end of Year 12. ^a^The timing of the CAMMS223 follow-up period, CAMMS03409 extension study, and TOPAZ extension study was variable, with most patients being followed up for 3 years, 4 years, and 2 years (to date), respectively, in each study; ^b^Patients had the option to receive additional alemtuzumab courses in the CAMMS223 follow-up period after the lifting of the dose suspension (in effect between 2005 and 2008); ^c^One patient was discontinued from the core CAMMS223 study and re-enrolled into the extension study. *AE* adverse event, *C* course, *DMT* disease-modifying therapy, *Y* year

CAMMS223 was an active-controlled, rater-blinded, head-to-head, 3-year trial of treatment-naive patients with early, active RRMS (Fig. 1). Early, active RRMS was defined as patients with an onset of symptoms ≤ 3 years prior to study entry, Expanded Disability Status Scale score (EDSS) of ≤ 3.0, ≥ 2 clinical episodes in the previous 2 years, and ≥ 1 gadolinium (Gd)-enhancing lesion on cranial magnetic resonance imaging (MRI). Patients were randomized to either alemtuzumab (two courses of 12 or 24 mg/day IV on 5 consecutive days at baseline and on 3 consecutive days 12 months later), or SC IFNB-1a (44 µg 3 times per week). A third course of alemtuzumab could be given ≥ 12 months after the last course at the treating physicians’ discretion if the CD4+ T-cell count was ≥ 100 × 10^6^ cells per liter [6]. Approximately 33 months from the study start, alemtuzumab dosing was suspended after three cases of ITP were reported, including one fatality, but efficacy and safety evaluations proceeded as planned [6].

#### CAMMS223 follow-up

After completion of the core CAMMS223 study, patients were invited to participate in an extended follow-up [17] (Fig. 1), the average duration of which was 2.5 years (standard deviation [SD], 1.0). In consultation with the investigator, after the dose suspension was lifted, patients had the option to receive additional alemtuzumab courses (12 mg/day on 3 consecutive days ≥ 12 months apart) during this follow-up period: two additional courses of alemtuzumab (not contingent upon evidence of disease activity) or alemtuzumab as needed for predefined disease activity (≥ 1 protocol-defined relapse or ≥ 2 Gd-enhancing or new/enlarging T2 hyperintense MRI lesions within the previous year).

#### CAMMS03409 extension study

On completion of this extended follow-up from the CAMMS223 study, patients were then eligible to enroll in the CAMMS03409 extension study and receive additional courses of alemtuzumab as needed for predefined disease activity at the investigator’s discretion [9–11] (Fig. 1).

#### TOPAZ extension study

Thereafter, further follow-up was available beyond CAMMS03409 as part of the TOPAZ extension study, where patients could again receive additional alemtuzumab at the discretion of the investigator (not contingent upon evidence of disease activity) [12, 13] (Fig. 1).

Treatment with other approved disease-modifying therapies (DMTs) was permitted at the investigator’s discretion during the CAMMS223 trial and its extended follow-up period, the CAMMS03409 extension, as well as the TOPAZ extension studies. The studies were conducted in accordance with the ethical principles outlined in the Declaration of Helsinki. All procedures were approved by local institutional ethics review boards of participating sites, and patients provided written informed consent.

### Assessments

Clinical efficacy assessments included annualized relapse rate (ARR), mean change from core study baseline in EDSS score, and proportions of patients with EDSS score stability (≤ 0.5-point change in either direction), improvement (≥ 1.0-point decrease), or worsening (≥ 1.0-point increase). Confirmed disability worsening (CDW) was defined as ≥ 1.0-point EDSS increase (or ≥ 1.5 points if baseline EDSS = 0) confirmed over 6 months, and confirmed disability improvement (CDI) was defined as ≥ 1.0-point EDSS decrease from baseline confirmed over 6 months (assessed only in patients with baseline EDSS ≥ 2.0).

MRI assessments included the proportions of patients free from new MRI lesions (new Gd-enhancing T1, new/enlarging T2 hyperintense, or new non-enhancing T1 hypointense lesions). Patients free of MRI disease activity were defined as having no new Gd-enhancing T1 lesions on current MRI or new/enlarging T2 hyperintense lesions since last MRI. MRI lesion counts were assessed only from the onset of the CAMMS03409 extension study and beyond.

No evidence of disease activity (NEDA) was defined as absence of both clinical disease activity (absence of both relapses and 6-month CDW) and MRI disease activity and was evaluated annually and cumulatively from the CAMMS03409 extension study enrollment and onward, until end of Year 12.

AEs, serious AEs, and AEs of special interest were recorded throughout the studies. IARs were defined as any AE with onset during or within 2 days after alemtuzumab infusion. Serious AEs were defined as fatal or life threatening, requiring or prolonging inpatient hospitalization, disabling, resulting in a congenital anomaly, or requiring medical or surgical intervention to influence these outcomes. AEs of special interest did not meet criteria for serious AEs but were of particular interest in the context of the study; these events included autoimmune cytopenias (i.e., ITP, autoimmune hemolytic anemia, and autoimmune neutropenia), glomerulonephritis, other autoimmune disorders, pregnancy, cervical dysplasia, opportunistic infections, and malignancies. Monitoring for autoimmune AEs included monthly complete blood cell count assessments and renal evaluations, and quarterly thyroid function testing for at least 4 years from the administration of the last alemtuzumab dose.

To examine the individual patients’ clinical course of MS at the time of study discontinuation, efficacy and safety outcomes were evaluated in alemtuzumab-treated patients from the CAMMS223 core study who did not enroll in the CAMMS03409 extension study.

### Statistics

This analysis included all available data (without imputation) for patients who received 12 mg/day alemtuzumab in the CAMMS223 core trial, enrolled in the CAMMS03409 extension study, with available further follow-up in TOPAZ. Patients who received 24 mg/day alemtuzumab in the core CAMMS223 study were excluded.

ARR was estimated through negative binomial regression with robust variance estimation. Proportions of patients with 6-month CDW or 6-month CDI events were determined with Kaplan–Meier estimates. MRI scans for Gd-enhancing lesions were not collected during the core CAMMS223 study and its follow-up period; hence, proportions of patients free of new MRI lesions and MRI disease activity, as well as proportions of patients with NEDA, were assessed from the onset of CAMMS03409 extension study, and evaluated annually onward. Proportions of patients with sustained NEDA, evaluated cumulatively from the onset of CAMMS03409 extension study until end of Year 12, were estimated with the Kaplan–Meier method.

Incidences (percentage of patients with ≥ 1 event) of AEs, serious AEs, and medical events of interest were reported annually.

In patients who withdrew prior to enrolling in the CAMMS03409 extension study, relapse rates and AEs were evaluated from core CAMMS223 study baseline (Year 0) to the time of discontinuation (Year 6). Disability outcomes (EDSS scores, 6-month CDW, and 6-month CDI) were assessed from core study baseline until the most recent EDSS assessment available prior to discontinuation (Year 5).

## Results

### Patient disposition and additional treatment

Of the 108 patients who received alemtuzumab 12 mg/day in the CAMMS223 core study, 60 (56%) enrolled in the CAMMS03409 extension study, and had further follow-up in TOPAZ; 54 (50%) patients remained on study at Year 12 (Fig. 1). Of the six patients who discontinued after enrolling in the extension study, three withdrew due to physician decision, two withdrew their consent, and one death occurred due to hemorrhagic shock from an accident unrelated to the study.

Over 12 years, 20/60 patients (33%) received a total of two courses of alemtuzumab, and 39/60 patients (65%) received additional courses, with 38%, 15%, 3%, 7%, and 2% receiving a total of 3, 4, 5, 6, and 7 courses, respectively. One patient (2%) received only one course. Of the 39 patients who received ≥ 3 alemtuzumab courses, 14 (36%) received a third course in the core CAMMS223 study, and 22 (56%) received it in Years 5–6; 3 patients (8%) received a third course in Years 7–9. Of the 16 patients who received ≥ 4 courses, 12 (75%) received a fourth course in Years 6–8, and 4 (25%) in Years 10–12. For most patients (57.4%), the reason for additional treatment was not documented. Among those for whom reasons were known, the most common were relapse activity (27.9%), MRI activity (4.4%), both relapse and MRI activity (8.8%), and both relapse and EDSS progression (1.5%). Through 12 years, 12/60 (20%) patients received other DMTs, most frequently interferons and glatiramer acetate.

### Efficacy in the long-term follow-up cohort

**Fig. 2:**
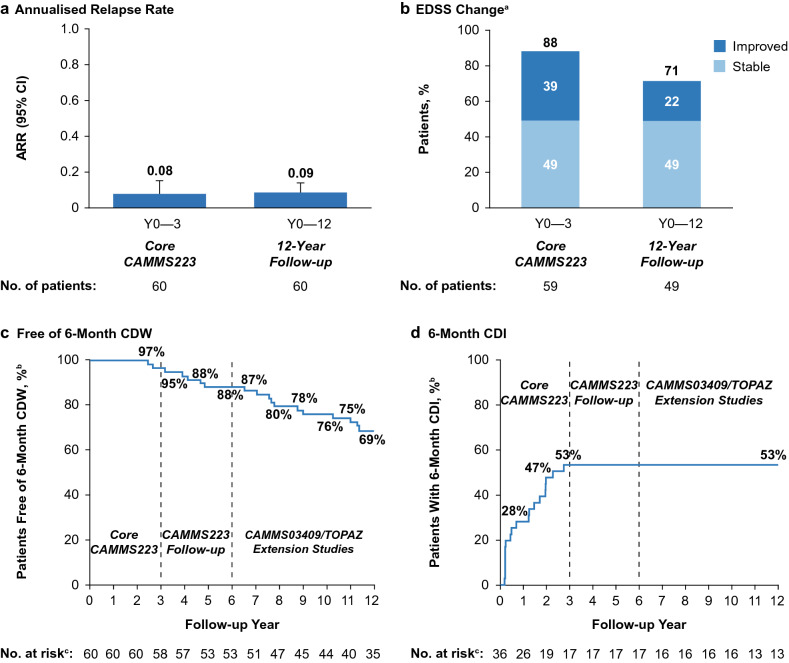
Clinical efficacy outcomes of the CAMMS223 long-term follow-up cohort over 12 years. ARR (**a**), proportions of patients with stable or improved EDSS scores (**b**), proportions of patients free of 6-month CDW (**c**), and proportions of patients achieving 6-month CDI (**d**) over 12 years in the long-term follow-up cohort. Error bars denote 95% CIs. ^a^Proportions of patients with stable (≤ 0.5-point change in either direction), or improved (≥ 1.0-point decrease) EDSS scores, assessed since core CAMMS223 study baseline. ^b^Kaplan–Meier estimates. ^c^Number at risk is the number of patients who remained on study and had yet to experience 6-month CDW or 6-month CDI. CDI is defined as ≥ 1-point EDSS decrease from baseline confirmed over 6 months (CDI is assessed only in patients with baseline EDSS score ≥ 2.0). CDW is defined as ≥ 1-point EDSS increase (or ≥ 1.5 points if baseline EDSS = 0) confirmed over 6 months. *ARR* annualized relapse rate, *CDI* confirmed disability improvement, *CDW* confirmed disability worsening, *CI* confidence interval, *EDSS* Expanded Disability Status Scale

Annually through Year 12, ARR remained low (0.03–0.14). The cumulative ARR was 0.09 over 12 years and similar to the relapse rate in the core CAMMS223 study (0.08; Fig. 2a). At Year 12, 49% of patients showed stable EDSS, and 22% showed improved EDSS from core study baseline (Fig. 2b). Mean EDSS change from baseline was + 0.33 (SD, 1.6). Through Year 12, 69% of patients were free of 6-month CDW (Fig. 2c), and 53% achieved 6-month CDI (Fig. 2d). To determine whether CDI was maintained over 12 years, patients who achieved CDI were assessed for subsequent CDW. Of the 19 patients who achieved CDI over 12 years, 17 (89%) had no subsequent CDW.

**Fig. 3:**
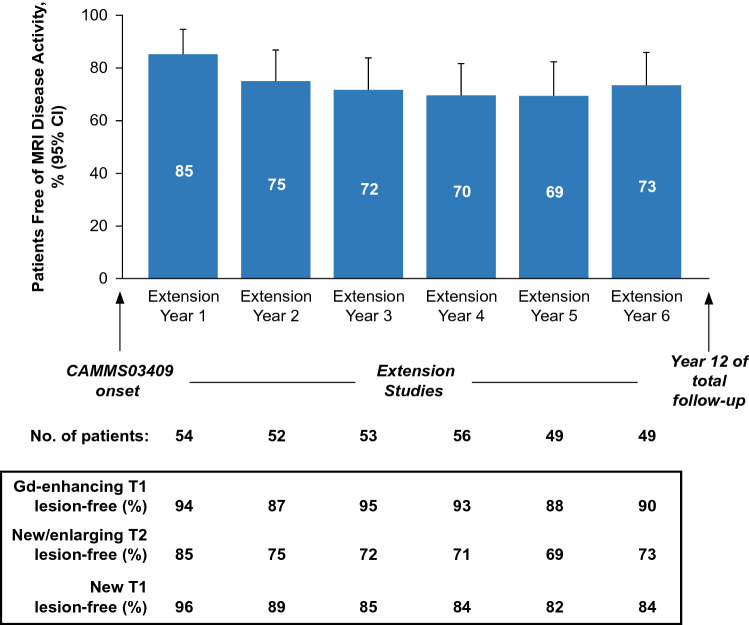
MRI lesion outcomes of the CAMMS223 long-term follow-up cohort. Proportions of patients free of MRI disease activity, and of new MRI lesions (Gd-enhancing T1, new/enlarging T2 and new T1) from Year 7 to Year 12 (CAMMS03409 extension study enrollment until end of Year 12) in the long-term follow-up cohort. MRI outcomes were assessed annually from the CAMMS03409 extension study enrollment and onward, until end of Year 12. Freedom from MRI disease activity was defined as the absence of new gadolinium-enhancing T1 and new/enlarging T2 hyperintense lesions. Error bars denote 95% CIs. *CI* confidence interval, *Gd* gadolinium

**Fig. 4:**
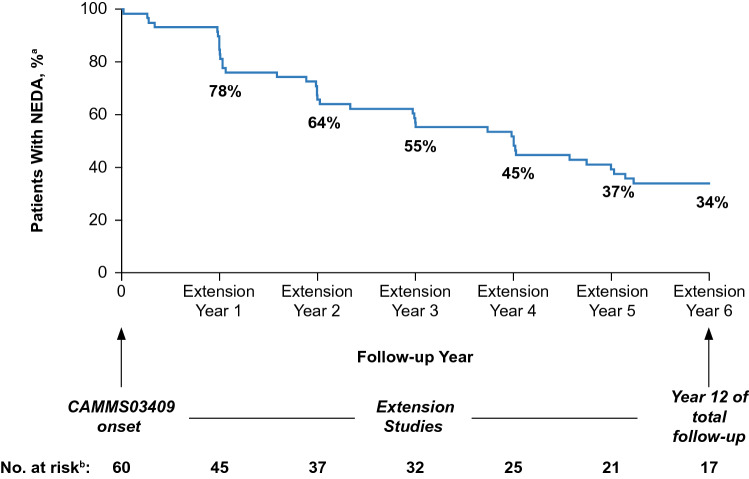
NEDA outcomes of the CAMMS223 long-term follow-up cohort over 12 years. Percentage of patients with NEDA from Year 7 to Year 12 (CAMMS03409 extension study enrollment until end of Year 12) in the long-term follow-up cohort. ^a^Kaplan–Meier estimates. ^b^Number at risk is the number of patients who remained on study and did not yet have disease activity. NEDA is defined as absence of both clinical disease activity (absence of both relapses and 6-month CDW) and MRI disease activity (absence of new gadolinium-enhancing T1 and new/enlarging T2 hyperintense lesions). *CDW* confirmed disability worsening, *CI* confidence interval, *NEDA* no evidence of disease activity

In each year of the CAMMS03409 and subsequent TOPAZ extension studies through Year 12, ≥ 69% of patients were free of MRI disease activity, with ≥ 87% having no new Gd-enhancing T1 lesions, and ≥ 69% having no new/enlarging T2 lesions; ≥ 82% were free of new T1 hypointense lesions (Fig. 3). Proportions of patients with annual NEDA were 63–78% through Year 12 (extension Year 1: 78%; extension Year 2: 67%; extension Year 3: 64%; extension Year 4: 68%; extension Year 5: 63%; extension Year 6: 67%). From the onset of the CAMMS03409 extension study and until the end of Year 12, 33.9% (95% CI, 22.0–46.1%) of patients had sustained NEDA (Fig. 4).

### Safety in the long-term follow-up cohort

AE incidences in the long-term follow-up cohort over 12 years are shown in Table 1. Incidence rates of most AEs declined in the CAMMS223 follow-up period and the CAMMS03409/TOPAZ extensions (Years 4–12) relative to the core study (Years 1–3). No patients withdrew from the extension studies due to AEs. The majority of AEs (97%) were mild to moderate. Nine grade 4 events occurred through 12 years: one event each of adhesive ileus and diffuse serious peritonitis (both in Year 1 in the same patient), two cases of neutropenia (one each in Years 4 and 5), two events of generalized rash (both in Year 1 in the same patient), and two malignant melanomas (one metastatic) and one lymphadenopathy (all in Year 10 in the same patient). One death occurred in Year 7 from a hemorrhagic shock due to deep left brachial vein damage as a result of an incised wound from an accident unrelated to the study.

**Table 1:** Incidence of AEs observed in the CAMMS223 long-term follow-up cohort through Year 12

	Incidence, %												EAIR per 100 patient-years^a^
	Y1 (*N* = 60)	Y2 (*N* = 60)	Y3 (*N* = 60)	Y4 (*N* = 60)	Y5 (*N* = 60)	Y6 (*N* = 60)	Y7 (*N* = 60)	Y8 (*N* = 58)	Y9 (*N* = 58)	Y10 (*N* = 57)	Y11 (*N* = 57)	Y12 (*N* = 52)	Y0–12 (*N* = 60)
Any AE	98.3	91.7	75.0	46.7	41.7	55.0	65.0	62.1	63.8	70.2	54.4	38.5	2321.5
Serious AEs	8.3	8.3	13.3	3.3	5.0	8.3	10.0	3.4	10.3	8.8	1.8	3.8	5.9
Infections	53.3	33.3	35.0	20.0	25.0	26.7	36.7	27.6	36.2	29.8	26.3	26.9	26.1
Serious infections	3.3	0	1.7	1.7	0	0	0	0	1.7	0	0	0	0.8
Autoimmune AEs^b^													
Thyroid AEs	1.7	15.0	15.0	6.7	1.7	1.7	3.3	0	1.7	0	3.5	0	6.6
Serious thyroid AEs	0	1.7	0	0	0	1.7	1.7	1.7	1.7	0	0	1.9	0.9
Immune thrombocytopenia	0	0	0	1.7	0	0	0	0	0	0	0	0	0.1
Nephropathies	0	0	0	0	0	0	0	0	0	0	0	0	0
Malignancies	0	0	0	0	0	0	0	0	0	3.5	0	0	0.3
Deaths	0	0	0	0	0	0	1.7	0	0	0	0	0	0.1

*AE* adverse event, *EAIR* exposure-adjusted incidence rate, *Y* year

^a^EAIR = (Number of patients with first AE in the time interval)/(Total follow-up duration [years] of all patients within the time interval, censoring at the time of AE for patients counted in the numerator) × 100

^b^First occurrence of AE for a patient

IARs declined after the first course of alemtuzumab (Course 1: 98%; Course 2: 80%; Course 3: 77%; Course 4: 63%). Of the 12 patients who received Courses 5 and 6, 7 (58%) reported IARs; no IARs were reported in the 1 patient who received Course 7. The most commonly reported IARs over 12 years were rash (65%) and headache (58%). In this long-term follow-up cohort, there were no reports of acute cardiovascular and pulmonary events, such as pulmonary alveolar hemorrhage, stroke, myocardial infarction, and cervicocephalic arterial dissection occurring shortly after initiation of alemtuzumab. Incidence of an ischemic stroke and a myocardial infarction was reported in 1 patient over 12 years, and occurred 1.9 and 1.8 years, respectively, after the most recent alemtuzumab dose.

Infection rate was highest during Year 1 (53%) and declined thereafter. Serious infections occurred in < 4% of patients during each year through Year 12. In 10% of patients, the first infection occurred within a year of the most recent alemtuzumab dose. There were no cases of *Listeria monocytogenes* or cytomegalovirus infection over 12 years.

Thyroid AEs peaked in Years 2 and 3 (15% in each), and subsequently declined. Five patients had thyroid AEs more than 4 years (range 4.0–8.8 years) after the last alemtuzumab course (four cases of hyperthyroidism/Graves’ disease [three were serious events] and one of Hashimoto’s thyroiditis). Over 12 years, there was one case of ITP, with an initial onset in Year 4 (16 months after the last alemtuzumab dose), which resolved spontaneously without treatment; the same patient experienced delayed ITP recurrence 8 years after the last alemtuzumab dose, which was treated with intravenous steroids and platelet transfusion [15]. There were no cases of autoimmune nephropathy observed over 12 years. One case of cholecystitis was observed in Year 10 and did not meet the defining criteria for acute acalculous cholecystitis. Four cases of grade 3/4 neutropenia were observed over 12 years, which occurred 11–46 months after the most recent alemtuzumab dose.

Two patients had malignancies; all were melanomas and occurred in Year 10 of follow-up. One patient with a family history of melanoma had two grade 4 malignant melanomas that were deemed by the study investigator to be possibly related to study drug. The other patient was diagnosed with grade 2 melanoma in situ (abdomen), considered unrelated to study drug by the investigator, and was resolved by surgical excision.

### Efficacy and safety in patients who discontinued prior to enrolling in the CAMMS03409 extension

Of the 48 patients who did not enroll in the CAMMS03409 extension study, 3 discontinued due to AEs, 2 due to lack of efficacy, and 1 death occurred due to cardiovascular disease [6]. Seventeen patients discontinued due to other reasons (including noncompliance with study drug, patient withdrawal, lost to follow-up, and physician decision), and no reason was provided for 25 patients (Fig. 1). Baseline characteristics were similar between patients who discontinued, and those that remained in the study through 12 years (Supplementary Table S1). Mean EDSS score in this group at the time of discontinuation was similar to the study start (1.9 vs. 2.0), and mean number of relapses in the 1–2 years prior to study withdrawal ranged from 0.3 to 0.4. At the time of withdrawal, cumulative ARR in discontinued patients was higher (0.19 in Years 0–6; Supplementary Fig. 1a) than in the long-term follow-up cohort (0.09 in Years 0–12; Fig. 2a). Disability outcomes in discontinued patients were generally consistent with the 12-year follow-up cohort. Over 5 years, 77% of patients showed stable or improved EDSS from core study baseline (Supplementary Fig. 1b). Through Year 5, 87% of patients remained free of 6-month CDW and 30% achieved 6-month CDI (Supplementary Fig. 1c, d). Safety findings in patients who discontinued were consistent with the long-term follow-up cohort, with AEs occurring most frequently in Year 1 (98%) and declining thereafter until discontinuation (Year 6). Thyroid AEs peaked at Year 3 (11.4%), with lower incidences observed through Years 4–6 (2.9–10.0%; Supplementary Table S2).

## Discussion

DMTs are effective in reducing relapses and early disability progression in patients with RRMS, but their long-term efficacy in various stages of MS is largely unknown [1]. Findings of our study show that alemtuzumab efficacy on clinical and MRI outcomes was maintained over 12 years in the long-term follow-up cohort of CAMMS223 patients who enrolled in the CAMMS03409 and the subsequent TOPAZ extension studies. ARR remained low over 12 years (0.09), and 69–85% of patients were free of MRI disease activity in each year of the extension studies. The majority (71%) of alemtuzumab-treated patients showed stable or improved disability based on EDSS scores through Year 12. Additionally, 34% of patients were free of disease activity over 6 years, from the onset of the CAMMS03409 extension study through end of Year 12, suggesting remission. Importantly, these results were achieved with 33% of patients receiving a total of 2 courses of alemtuzumab, and 73% receiving no more than three courses over 12 years. These results are consistent with the 9-year analysis of the phase 3 CARE-MS studies, in which improvements were seen in clinical, MRI, and disability outcomes in alemtuzumab-treated RRMS patients [12, 13].

Patient retention in long-term trials over multiple years can be challenging; personal reasons or fatigue due to study burden can contribute to high discontinuation rates [18]. In our study, 50% of patients originally randomized to the core CAMMS223 trial remained on study through 12 years. This is higher than the retention rate reported for patients on fingolimod in the LONGTERMS extension study, where 41% of the 3168 patients enrolled have received more than 2 years of treatment; only a small cohort of 25 patients has received treatment for up to 10 years [19]. While higher retention rates (56–71%) have been reported in extension studies with other DMTs such as cladribine, natalizumab, and ocrelizumab, to date these have been over a shorter follow-up period ranging from 4 to 5 years [20–22].

The most common reason for discontinuation in our study was noncompliance with study drug, and nearly all patients who discontinued for this reason did so during the CAMMS223 core trial. Other reasons were likely related to the length of the trial, and included patient withdrawal, failure to maintain follow-up, and physician decision; 23% of the originally CAMMS223 randomized cohort withdrew with no reason provided. Notably, discontinuations due to documented lack of efficacy or presence of AEs were low. In that respect, baseline characteristics of CAMMS223 patients who discontinued prior to enrollment in the CAMMS03409 extension were similar to the long-term follow-up cohort who remained on study through 12 years. Compared with the 12-year cohort, patients who discontinued had higher ARR at the time of study withdrawal (0.09 vs. 0.19). However, other clinical efficacy outcomes up to the time of discontinuation were largely similar between the two cohorts, arguing against a selection bias in favor of continuation in patients who perceived themselves to be benefitting from treatment or withdrawal in others who may have been dissatisfied with their treatment.

The accumulation of impairment and disability over the long-term has a profound negative impact on MS patients. Thus, reducing disease progression and improving preexisting disability is an important treatment goal. Our results showed that 71% of alemtuzumab-treated patients had improved or stable EDSS scores over 12 years, and the average EDSS score change from baseline to Year 12 was + 0.33. Nearly 70% of patients were free of 6-month CDW, and 53% attained 6-month CDI. Of those that achieved CDI at any time during the course of the study, 89% were free from CDW, indicating that improvement in preexisting disability was consistent and generally maintained over 12 years.

Most patients (73%) in this 12-year cohort required no more than one additional alemtuzumab course beyond the initial two courses as prescribed in the protocol. Patients most frequently received additional treatment with alemtuzumab in the later years, providing further support for maintenance of efficacy over the long-term. Although the exact mechanism of action is not fully known, the sustained treatment effect with alemtuzumab may be the result of immunomodulatory effects due to the selective depletion of circulating T and B lymphocytes and with a subsequent distinct pattern of lymphocyte repopulation following treatment, which leads to a shift from pro- to anti-inflammatory cytokine profile [23–26]. Other studies have suggested that the impact of alemtuzumab may extend beyond its anti-inflammatory effect and include neuroprotection through stimulation of neurotrophin production, which may contribute to the sustained improvements in disability observed over the long term [27].

Safety findings of the CAMMS223 12-year cohort were consistent with prior phase 2, phase 3, and extension studies [6–10, 17]. IARs were most frequent at the first alemtuzumab course but declined with subsequent courses. Overall AEs, including infections, were most prevalent in the first year of the study with reduced incidences in the extension studies compared with the core trial. Notably, life-threatening infections, in particular *Listeria monocytogenes* or cytomegalovirus infection, were not observed in this 12-year cohort. Thyroid AEs, the most commonly occurring autoimmune events, peaked in Years 2 and 3 of the core trial and declined thereafter, consistent with the trend seen in 9-year analyses of patients from the phase 3 CARE-MS studies [12, 13]. In this 12-year cohort of CAMM223 patients, one case of ITP occurred in Year 4 (within 4 years since last alemtuzumab dose) with no new cases in Years 5–12. There were no cases of autoimmune nephropathies over 12 years. Five cases of thyroid AEs (including three serious events) were reported after 4 years since the last alemtuzumab course. A comprehensive risk management strategy including physician and patient education combined with the prescribed safety monitoring program has helped minimize the treatment risks with alemtuzumab [28, 29]. With the emergence of additional AEs observed during post-marketing use of alemtuzumab, including those occurring shortly after infusion, the European Medicines Agency (EMA) has recently recommended alemtuzumab to be used only in patients with highly active disease (despite treatment with ≥ 1 DMT) or rapidly worsening disease, and without certain heart, circulation or bleeding disorders or autoimmune disorders other than MS [30]. It is worth noting that none of these new safety signals, including possibly infusion-related serious side effects or life-threatening autoimmune diseases such as autoimmune hepatitis and hemophagocytic lymphohistiocytosis, were observed in this 12-year cohort.

The relatively small sample size of the 12-year cohort is a limitation of this analysis. Additionally, a selection bias in favor of patients with a positive experience with alemtuzumab has to be considered, since patients participated in the extension study voluntarily. However, apart from a slightly higher ARR, the efficacy and safety outcomes in patients who discontinued prior to the start of the extension study were largely consistent with those who remained in the study through 12 years, and may have minimized the potential for selection bias.

## Conclusions

In summary, our results show that efficacy of alemtuzumab on clinical and MRI outcomes was maintained over 12 years in the long-term follow-up of a cohort of patients from the CAMMS223 trial who were treatment naive at study entry and who enrolled in extension studies. This was achieved with 33% of patients receiving a total of two courses of alemtuzumab through Year 12, and 73% receiving no more than three courses. The safety profile of alemtuzumab in this long-term follow-up cohort over the 12-year time-frame was consistent with other alemtuzumab clinical trials.

## Electronic supplementary material

Below is the link to the electronic supplementary material.

Supplementary file1 (DOCX 67 kb)

Supplementary file2 (DOCX 71 kb)

Supplementary file3 (EPS 1180 kb)

## Data Availability

Qualified researchers may request access to patient-level data and related study documents including the clinical study report, study protocol with any amendments, blank case report form, statistical analysis plan, and dataset specifications. Patient-level data will be anonymized and study documents will be redacted to protect the privacy of trial participants. Further details on Sanofi’s data sharing criteria, eligible studies, and process for requesting access can be found at https://www.clinicalstudydatarequest.com.
