# Supplementary material for: Long-term efficacy and safety of alemtuzumab in patients with RRMS: 12-year follow-up of CAMMS223
Source: J Neurol. 2020 Jun 24;267(11):3343–53. doi: 10.1007/s00415-020-09983-1 (PMC7578137; doi:10.1007/s00415-020-09983-1)
Supplement: Supplementary file 1 — Supplementary file1 (DOCX 67 kb) [file 415_2020_9983_MOESM1_ESM.docx]

**Supplementary Figure Legends**

**Article title:** Long-term efficacy and safety of alemtuzumab in patients with RRMS: 12-year follow-up of CAMMS223 **Journal:** *Journal of Neurology*

**Authors:** Brian Steingo, Yaser Al Malik, Ann D. Bass, Regina Berkovich, Matthew Carraro, Óscar Fernández, Carolina Ionete, Luca Massacesi, Sven G. Meuth, Dimos D. Mitsikostas, Gabriel Pardo, Renata Faria Simm, Anthony Traboulsee, Zia Choudhry, Nadia Daizadeh, D Alastair S. Compston; on behalf of the CAMMS223, CAMMS03409, and TOPAZ investigators

**Correspondence to:**

Brian Steingo

Infinity Clinical Research

3537 N. Pine Island Road,

Sunrise, FL 33351Email: ogniets@yahoo.com

Tel: +1 954-475-4811

Fax: +1 954-472-0556

**Supplementary Fig. 1** Clinical efficacy outcomes of the CAMMS223 patient cohort who did not enroll in the CAMMS03409 extension study.

ARR (**a**), proportions with stable or improved EDSS scores (**b**), proportions free of 6-month CDW (**c**), and proportions achieving 6-month CDI (**d**) in the cohort of patients who were randomised to 12 mg alemtuzumab in the CAMMS223 trial, and discontinued prior to enrollment in the CAMMS03409 extension study. Relapse rates were assessed from core CAMMS223 study baseline (Year 0) to the time of discontinuation (Year 6). Disability outcomes (EDSS scores, 6-month CDW, and 6-month CDI) were assessed from core CAMMS223 study baseline until the most recent EDSS assessment available prior to discontinuation (Year 5). Error bars denote 95% CIs. ^a^Proportions of patients with stable (≤ 0.5-point change in either direction), or improved (≥ 1.0-point decrease) EDSS scores, assessed since core CAMMS223 study baseline. ^b^Kaplan-Meier estimates. ^c^Number at risk is the number of patients who remained on study and had yet to experience 6-month CDW or 6-month CDI. CDI is defined as ≥ 1-point EDSS decrease from baseline confirmed over 6 months (CDI is assessed only in patients with baseline EDSS score ≥ 2.0). CDW is defined as ≥ 1-point EDSS increase (or ≥1.5 points if baseline EDSS = 0) confirmed over 6 months. *ARR* annualised relapse rate, *CDI* confirmed disability improvement, *CDW* confirmed disability worsening, *CI* confidence interval, *EDSS* Expanded Disability Status Scale
