# Supplementary material for: Long-term efficacy and safety of alemtuzumab in patients with RRMS: 12-year follow-up of CAMMS223
Source: J Neurol. 2020 Jun 24;267(11):3343–53. doi: 10.1007/s00415-020-09983-1 (PMC7578137; doi:10.1007/s00415-020-09983-1)
Supplement: Supplementary file 2 — Supplementary file2 (DOCX 71 kb) [file 415_2020_9983_MOESM2_ESM.docx]

**Supplementary Tables**

**Article title:** Long-term efficacy and safety of alemtuzumab in patients with RRMS: 12-year follow-up of CAMMS223 **Journal:** *Journal of Neurology*

**Authors:** Brian Steingo, Yaser Al Malik, Ann D. Bass, Regina Berkovich, Matthew Carraro, Óscar Fernández, Carolina Ionete, Luca Massacesi, Sven G. Meuth, Dimos D. Mitsikostas, Gabriel Pardo, Renata Faria Simm, Anthony Traboulsee, Zia Choudhry, Nadia Daizadeh, D Alastair S. Compston; on behalf of the CAMMS223, CAMMS03409, and TOPAZ investigators

**Correspondence to:**

Brian Steingo

Infinity Clinical Research

3537 N. Pine Island Road,

Sunrise, FL 33351Email: ogniets@yahoo.com

Tel: +1 954-475-4811

Fax: +1 954-472-0556

**Supplementary Table S1** Baseline characteristics of the CAMMS223 long-term follow-up cohort and of CAMMS223 patients who discontinued prior to enrolling in the CAMMS03409 extension study

| Parameter | Alemtuzumab 12 mg | |
| --- | --- | --- |
|  | Long-term  follow-up cohort (*N*=60) | Withdrawn prior to CAMMS03409 enrollment  (*N*=48) |
| Age, years | 32.5 (8.5) | 32.1 (7.5) |
| Female, *n* (%) | 34 (56.7) | 36 (75.0) |
| EDSS score | 2.0 (0.7) | 2.0 (0.8) |
| Years since initial relapse | 1.3 (0.8) | 1.2 (0.8) |
| No. of relapses in prior 1 year | 1.9 (0.8) | 1.7 (0.9) |
| No. of relapses in prior 2 years | 2.6 (0.8) | 2.3 (0.9) |

All values are mean (standard deviation) unless indicated otherwise. *EDSS* Expanded Disability Status Scale

**Supplementary Table S2** Incidence of AEs observed in the CAMMS223 patient cohort who discontinued prior to enrolling in the CAMMS03409 extension study

|  | Incidence, % | | | | | | EAIR per 100 patient-years^a^ | |
| --- | --- | --- | --- | --- | --- | --- | --- | --- |
|  | Y1  (*N*=48) | Y2  (*N*=48) | Y3  (*N*=44) | Y4  (*N*=34) | Y5  (*N*=20) | Y6  (*N*=14) | Y0–6  (*N*=48) |  |
| Any AE | 97.9 | 89.6 | 86.4 | 35.3 | 60.0 | 21.4 | 3667.8 |  |
| Serious AEs | 2.1 | 6.3 | 15.9 | 2.9 | 15.0 | 0 | 7.6 |  |
| Infections | 43.8 | 33.3 | 34.1 | 11.8 | 35.0 | 7.1 | 41.6 |  |
| Serious infections | 0 | 0 | 2.3 | 0 | 5.0 | 0 | 1.1 |  |
| Autoimmune AEs^b^ | | | | | | |  |  |
| Thyroid AEs | 8.3 | 4.2 | 11.4 | 2.9 | 10.0 | 0 | 9.2 |  |
| Serious thyroid AEs | 0 | 0 | 0 | 2.9 | 0 | 0 | 0.6 |  |
| Immune thrombocytopenia | 0 | 0 | 2.3 | 0 | 0 | 0 | 0.6 |  |
| Nephropathies | 0 | 0 | 0 | 0 | 0 | 0 | 0 |  |
| Malignancies | 0 | 0 | 0 | 0 | 0 | 0 | 0 |  |
| Deaths | 0 | 0 | 0 | 0 | 0 | 0 | 0 |  |

AE incidences were reported from beginning of CAMMS223 core study until time of discontinuation prior to CAMMS03409 onset. ^a^EAIR=(Number of patients with first AE in the time interval)/(Total follow-up duration [years] of all patients within the time interval, censoring at the time of AE for patients counted in the numerator) × 100; ^b^First occurrence of AE for a patient. *AE* adverse event, *EAIR* exposure-adjusted incidence rate
